# Cancer Distribution Among Asian, Native Hawaiian, and Pacific Islander Subgroups — United States, 2015–2019

**DOI:** 10.15585/mmwr.mm7216a2

**Published:** 2023-04-21

**Authors:** Suzanne Bock, S. Jane Henley, Mary Elizabeth O’Neil, Simple D. Singh, Trevor D. Thompson, Manxia Wu

**Affiliations:** 1 Division of Cancer Prevention and Control, National Center for Chronic Disease Prevention and Health Promotion, CDC.

Non-Hispanic Asian (Asian) and non-Hispanic Native Hawaiian and Pacific Islander (NHPI) persons represent growing segments of the U.S. population (*1*). Epidemiologic cancer studies often aggregate Asian and NHPI persons (*2*,*3*); however, because Asian and NHPI persons are culturally, geographically, and linguistically diverse (*2*,*4*), subgroup analyses might provide insights into the distribution of health outcomes. To examine the frequency and percentage of new cancer cases among 25 Asian and NHPI subgroups, CDC analyzed the most current 2015–2019 U.S. Cancer Statistics data.* The distribution of new cancer cases among Asian and NHPI subgroups differed by sex, age, cancer type, and stage at diagnosis (for screening-detected cancers). The percentage of cases diagnosed among females ranged from 47.1% to 68.2% and among persons aged <40 years, ranged from 3.1% to 20.2%. Among the 25 subgroups, the most common cancer type varied. For example, although breast cancer was the most common in 18 subgroups, lung cancer was the most common cancer among Chamoru, Micronesian race not otherwise specified (NOS), and Vietnamese persons; colorectal cancer was the most common cancer among Cambodian, Hmong, Laotian, and Papua New Guinean persons. The frequency of late-stage cancer diagnoses among all subgroups ranged from 25.7% to 40.3% (breast), 38.1% to 61.1% (cervical), 52.4% to 64.7% (colorectal), and 70.0% to 78.5% (lung). Subgroup data illustrate health disparities among Asian and NHPI persons, which might be reduced through the design and implementation of culturally and linguistically responsive cancer prevention and control programs, including programs that address social determinants of health.

Invasive cancer cases were defined according to the World Health Organization *International Classification of Diseases for Oncology, Third Edition*^†^ diagnosed during 2015–2019 using the most current U.S. Cancer Statistics data. This source of high-quality incidence data from population-based cancer registries, supported by CDC’s National Program of Cancer Registries and the National Cancer Institute’s Surveillance, Epidemiology, and End Results Program, covers approximately 99% of the U.S. population during the 5-year period.

Central cancer registries collect race and ethnicity information from different sources, including self-reported intake questionnaires, abstracted patient records, electronic health records, linkages to administrative databases, and algorithms to impute missing data (*5*). The current analysis is restricted to Asian and NHPI persons who reported non-Hispanic ethnicity. Race was recorded by standardized coding methods using 30 race groups, including 25 Asian and NHPI subgroups.^§^ Some subgroups were defined by region rather than race (e.g., Micronesian race NOS). Because of low case counts, in some analyses, Cambodian, Hmong, Laotian, and Thai persons were aggregated into an Other Southeast Asian group.^¶^ Data for other racial groups are available in the Data Visualizations Tool** (Supplementary Table, https://stacks.cdc.gov/view/cdc/126010). Cases were stratified by race, sex, and age for all cancers combined and then categorized into the 10 most common cancer types among all Asian and NHPI persons. A subset of cancer types detectable by screening^††^ (i.e., female breast, colon and rectum, lung and bronchus, and cervix uteri) were further categorized by stage at diagnosis as early-stage, late-stage, or unknown.^§§^ Because current national population denominators are not available for all subgroups, results are presented as frequencies and percentages rather than rates. In all analyses, cells containing fewer than six cases were suppressed to protect confidentiality and reduce misinterpretation or misuse of unstable counts. This activity was reviewed by CDC and was conducted consistent with applicable federal law and CDC policy.^¶¶^

During 2015–2019, a total of 273,656 new invasive cancer cases were reported among Asian persons and 18,491 among NHPI persons in the United States (Table 1); these included 92,562 in East Asian persons (31.7% of all cases among Asian and NHPI persons), 71,721 in Southeast Asian persons (24.5%), 44,890 in South Asian persons (15.4%), and 64,483 in Other Asian persons (22.1%). Approximately one half of cases among Asian (56.2%) and NHPI (56.5%) persons were diagnosed in females, and approximately one tenth were diagnosed in persons aged <40 years (including 8.5% in Asian and 9.6% in NHPI persons). Across subgroups, the highest percentages of new cancer cases among females occurred among Tahitian (68.2%), Thai (65.5%), and Fiji Islander (65.1%) subgroups. The percentage of new cancer cases in persons aged <40 years was highest among Hmong (20.2%), Micronesian race NOS (18.1%), and Melanesian race NOS persons (15.7%), and lowest among Japanese persons (3.1%).

**TABLE 1:** Cancer diagnosis stratified by sex and age groups, by race and ethnicity — United States, 2015–2019*

Race and ethnicity^†^	No. (%)^§,¶^						
	Total**	Sex		Age group at diagnosis, yrs			
		Male	Female	<40	40–64	65–74	≥75
**Asian American, Native Hawaiian, or Pacific Islander**	**292,147 (100.0)**	**127,857 (43.8)**	**164,290 (56.2)**	**25,054 (8.6)**	**127,096 (43.5)**	**74,837 (25.6)**	**65,148 (22.3)**
**Asian American**	**273,656 (93.7)**	119,822 (43.8)	153,834 (56.2)	23,272 (8.5)	118,380 (43.3)	69,925 (25.6)	62,079 (22.7)
**East Asian**	**92,562 (31.7)**	40,862 (44.1)	51,700 (55.9)	5,007 (5.4)	35,946 (38.8)	23,358 (25.2)	28,251 (30.5)
Chinese	**55,181 (18.9)**	25,181 (45.6)	30,000 (54.4)	3,537 (6.4)	22,831 (41.4)	13,581 (24.6)	15,232 (27.6)
Japanese	**18,467 (6.3)**	7,565 (41.0)	10,902 (59.0)	564 (3.1)	5,505 (29.8)	4,804 (26.0)	7,594 (41.1)
Korean	**18,914 (6.5)**	8,116 (42.9)	10,798 (57.1)	906 (4.8)	7,610 (40.2)	4,973 (26.3)	5,425 (28.7)
**Southeast Asian**	**71,721 (24.5)**	30,689 (42.8)	41,032 (57.2)	4,445 (6.2)	31,759 (44.3)	19,934 (27.8)	15,583 (21.7)
Cambodian	**2,198 (0.8)**	1,021 (46.5)	1,177 (53.5)	184 (8.4)	1,008 (45.9)	595 (27.1)	411 (18.7)
Filipino	**42,330 (14.5)**	16,208 (38.3)	26,122 (61.7)	2,446 (5.8)	17,970 (42.5)	12,296 (29.0)	9,618 (22.7)
Hmong	**877 (0.3)**	365 (41.6)	512 (58.4)	177 (20.2)	346 (39.5)	161 (18.4)	193 (22.0)
Laotian	**1,918 (0.7)**	1,015 (52.9)	903 (47.1)	124 (6.5)	980 (51.1)	480 (25.0)	334 (17.4)
Thai	**2,287 (0.8)**	789 (34.5)	1,498 (65.5)	227 (9.9)	1,023 (44.7)	747 (32.7)	290 (12.7)
Vietnamese	**22,111 (7.6)**	11,291 (51.1)	10,820 (48.9)	1,287 (5.8)	10,432 (47.2)	5,655 (25.6)	4,737 (21.4)
**South Asian**	**44,890 (15.4)**	21,016 (46.8)	23,874 (53.2)	5,826 (13.0)	20,286 (45.2)	11,226 (25.0)	7,552 (16.8)
Asian Indian	**22,863 (7.8)**	10,781 (47.2)	12,082 (52.8)	3,020 (13.2)	9,917 (43.4)	5,794 (25.3)	4,132 (18.1)
Pakistani	**3,025 (1.0)**	1,450 (47.9)	1,575 (52.1)	329 (10.9)	1,496 (49.5)	796 (26.3)	404 (13.4)
Asian Indian or Pakistani, race NOS	**19,002 (6.5)**	8,785 (46.2)	10,217 (53.8)	2,477 (13.0)	8,873 (46.7)	4,636 (24.4)	3,016 (15.9)
**Other Asian**	**64,483 (22.1)**	27,255 (42.3)	37,228 (57.7)	7,994 (12.4)	30,389 (47.1)	15,407 (23.9)	10,693 (16.6)
**Native Hawaiian or Pacific Islander**	**18,491 (6.3)**	8,035 (43.5)	10,456 (56.5)	1,782 (9.6)	8,716 (47.2)	4,912 (26.6)	3,069 (16.6)
Chamoru	**132 (0)**	69 (52.3)	63 (47.7)	13 (9.8)	61 (46.2)	37 (28.0)	21 (15.9)
Fiji Islander	**364 (0.1)**	127 (34.9)	237 (65.1)	30 (8.2)	200 (54.9)	85 (23.4)	49 (13.5)
Guamanian, race NOS	**301 (0.1)**	132 (43.9)	169 (56.1)	27 (9.0)	134 (44.5)	90 (29.9)	50 (16.6)
Melanesian, race NOS	**51 (0)**	25 (49.0)	26 (51.0)	8 (15.7)	24 (47.1)	12 (23.5)	7 (13.7)
Micronesian, race NOS	**731 (0.3)**	319 (43.6)	412 (56.4)	132 (18.1)	393 (53.8)	153 (20.9)	53 (7.3)
Native Hawaiian	**10,668 (3.7)**	4,643 (43.5)	6,025 (56.5)	898 (8.4)	4,761 (44.6)	3,014 (28.3)	1,995 (18.7)
Pacific Islander, race NOS	**4,040 (1.4)**	1,757 (43.5)	2,283 (56.5)	471 (11.7)	2,027 (50.2)	952 (23.6)	590 (14.6)
Papua New Guinean	**48 (0)**	24 (50.0)	24 (50.0)	—^††^	21 (43.8)	—	—
Polynesian, race NOS	**123 (0)**	45 (36.6)	78 (63.4)	12 (9.8)	60 (48.8)	33 (26.8)	18 (14.6)
Samoan	**1,512 (0.5)**	665 (44.0)	847 (56.0)	159 (10.5)	767 (50.7)	378 (25.0)	208 (13.8)
Tahitian	**22 (0)**	7 (31.8)	15 (68.2)	—	14 (63.6)	—	—
Tongan	**499 (0.2)**	222 (44.5)	277 (55.5)	32 (6.4)	254 (50.9)	146 (29.3)	67 (13.4)

**Abbreviation**: NOS = not otherwise specified.

* Cancer incidence data were compiled from registries that meet the data quality criteria for all invasive cancer sites combined, representing 99% of the U.S. population.

^†^ The current analysis is restricted to persons who reported non-Hispanic ethnicity.

^§^ The denominator used to calculate percentages of new cancer cases among Asian American, Native Hawaiian, and Pacific Islander population is 292,147.

^¶^ Percentage calculated using each racial group's total number of cases as a denominator.

** Counts from suppressed cells are included in the aggregate totals.

^††^ Dashes indicate that counts were suppressed because fewer than six cases were reported or for complementary cell suppression.

Breast cancer accounted for the highest proportion of new cancer diagnoses among 18 (72.0%) of the 25 Asian and NHPI subgroups. Lung cancer was the most common cancer among Chamoru, Micronesian race NOS, and Vietnamese persons; colorectal cancer was the most common cancer among Cambodian, Hmong, Laotian, and Papua New Guinean persons (Table 2) (Supplementary Table, https://stacks.cdc.gov/view/cdc/126010).

**TABLE 2:** Percentage* of new invasive cancer diagnoses, by 10 common cancer types and race and ethnicity — United States, 2015–2019^†^

Race and ethnicity^§^	Cancer site, %									
	Female breast	Lung and bronchus	Colon and rectum	Prostate	Thyroid	Non-Hodgkin lymphoma	Liver and intrahepatic bile duct	Corpus uteri and uterus NOS	Stomach	Pancreas
**Asian American, Native Hawaiian, or Pacific Islander**	19.3	11.2	9.9	8.6	5.4	4.4	4.1	4.1	3.1	3.1
**Asian American**	19.3	11.2	10.0	8.6	5.5	4.4	4.2	3.9	3.1	3.1
**East Asian**	17.8	13.9	10.9	7.6	4.2	4.1	4.4	3.1	4.8	3.9
Chinese	16.9	15.3	10.2	7.3	4.9	4.1	4.7	3.2	4.2	3.5
Japanese	20.5	11.4	11.8	10.3	1.9	4.7	3.1	3.3	3.6	4.9
Korean	17.9	12.1	12.1	5.7	4.5	3.5	4.9	2.3	7.7	4.2
**Southeast Asian**	19.7	13.3	10.1	7.4	4.9	4.3	5.8	4.4	2.3	3.1
Cambodian	14.8	12.3	15.9	3.7	4.9	5.0	11.0	2.0	2.2	3.3
Filipino	23.4	11.7	9.0	8.8	5.4	4.3	3.1	5.5	1.6	3.3
Hmong	9.0	9.7	13.1	1.4	3.1	4.6	7.9	4.6	6.2	4.4
Laotian	11.7	14.1	14.8	4.1	2.0	4.5	11.7	2.6	3.0	3.7
Thai	22.5	13.1	9.7	6.1	4.5	4.2	4.7	4.3	2.6	2.5
Vietnamese	13.9	16.4	11.2	5.8	4.3	4.4	9.9	2.7	3.4	2.8
**South Asian**	20.7	6.9	8.0	9.7	6.3	4.7	2.8	4.1	2.2	2.5
Asian Indian	20.7	6.5	7.5	9.5	5.9	4.8	2.5	4.3	2.2	2.4
Pakistani	20.6	6.9	7.5	7.7	4.7	5.1	5.6	4.2	1.9	3.1
Asian Indian or Pakistani, race NOS	20.6	7.3	8.8	10.3	7.0	4.6	2.7	3.9	2.3	2.5
**Other Asian**	20.2	7.9	9.8	10.6	7.5	4.7	3.1	4.4	2.3	2.3
**Native Hawaiian or Pacific Islander**	18.6	10.9	8.9	9.3	4.1	3.8	3.2	7.4	2.5	3.1
Chamoru	13.6	18.9	13.6	4.5	—^¶^	—	—	—	5.3	—
Fiji Islander	27.5	6.1	8.8	5.8	2.5	4.1	1.9	7.7	3.9	4.1
Guamanian, race NOS	17.3	14.0	9.7	7.0	3.0	4.0	6.3	6.7	—	4.7
Melanesian, race NOS	25.5	—	—	13.7	—	—	—	—	—	—
Micronesian, race NOS	11.5	11.7	3.7	9.6	4.9	5.1	5.2	8.2	3.7	2.2
Native Hawaiian	19.4	11.5	9.2	9.2	4.1	3.7	3.1	6.5	2.0	3.3
Pacific Islander, race NOS	19.0	8.7	8.9	10.8	5.2	4.3	2.6	7.1	2.2	2.3
Papua New Guinean	14.9	12.8	14.9	—	—	—	—	—	—	—
Polynesian, race NOS	13.8	10.6	7.3	—	—	—	—	17.9	—	—
Samoan	13.9	12.8	9.0	8.1	2.3	3.3	3.2	12.8	4.7	3.8
Tahitian	27.3	—	—	—	—	—	—	—	—	—
Tongan	18.1	10.0	6.6	8.4	2.0	4.0	6.6	12.7	3.6	3.2

**Abbreviation**: NOS = not otherwise specified.

* Percentage was calculated by dividing the number of new cancer cases for 10 common cancer types by the total number of cancer cases in each racial group. The cancer types are based on the 10 most common cancer types diagnosed among all Asian American, Native Hawaiian, and Pacific Islander persons combined.

^†^ Cancer incidence data were compiled from registries that meet the data quality criteria for all invasive cancer sites combined, representing 99% of the U.S. population.

^§^ The current analysis is restricted to persons who reported non-Hispanic ethnicity.

^¶^ Dashes indicate that counts were suppressed because fewer than six cases were reported.

Among Asian and NHPI subgroups, the frequency of late-stage diagnoses for screening-detected cancers ranged from 25.7% (Japanese) to 40.2% (Other Southeast Asian) and 40.3% (Pacific Islander) for breast cancer; from 38.1% (Other Asian) to 61.1% (Korean) for cervical cancer; from 52.4% (Other Asian) to 64.7% (Other Southeast Asian) for colorectal cancer; and from 70.0% (Other Asian) to 78.5% (Other Southeast Asian) for lung cancer (Table 3).

**TABLE 3:** Percentage of four invasive cancers detectable by screening, by race and ethnicity and stage* at diagnosis — United States,^†^ 2015–2019

Race and ethnicity^§^ and stage	Female breast, %	Colon and rectum,%	Lung and bronchus, %	Cervix uteri, %
**Asian American, Native Hawaiian, or Pacific Islander**				
Early-stage	65.3	34.3	22.5	45.7
Late-stage	32.5	59.0	72.9	48.6
Unknown	2.2	6.7	4.6	5.7
**Asian American**				
Early-stage	65.6	34.2	22.6	45.9
Late-stage	32.3	59.0	72.8	48.3
Unknown	2.2	6.8	4.6	5.8
**East Asian**				
**Chinese**				
Early-stage	68.2	34.4	25.0	48.3
Late-stage	30.0	58.6	70.5	46.2
Unknown	1.7	7.0	4.4	5.5
**Japanese**				
Early-stage	72.9	33.8	20.4	42.4
Late-stage	25.7	60.5	73.3	51.4
Unknown	1.4	5.7	6.4	6.3
**Korean**				
Early-stage	64.1	30.3	21.2	35.4
Late-stage	33.8	63.1	73.8	61.1
Unknown	2.1	6.7	5.0	3.5
**Southeast Asian**				
**Filipino**				
Early-stage	64.8	32.3	22.0	41.1
Late-stage	33.5	61.8	73.9	55.1
Unknown	1.8	5.9	4.2	3.8
**Vietnamese**				
Early-stage	64.9	30.9	18.9	43.4
Late-stage	32.9	62.9	77.3	51.3
Unknown	2.2	6.1	3.8	5.3
**Other Southeast Asian** ^¶^				
Early-stage	57.5	28.9	16.6	42.2
Late-stage	40.2	64.7	78.5	50.0
Unknown	2.4	6.4	4.9	7.8
**South Asian** ^¶^				
Early-stage	61.9	33.7	21.7	42.2
Late-stage	35.9	60.6	73.8	51.0
Unknown	2.1	5.7	4.4	6.7
**Other Asian** ^¶^				
Early-stage	66.0	39.1	25.0	54.6
Late-stage	31.0	52.4	70.0	38.1
Unknown	3.0	8.5	5.0	7.2
**Native Hawaiian or Pacific Islander**				
Early-stage	61.4	35.2	20.3	43.2
Late-stage	35.7	59.1	75.3	52.3
Unknown	2.9	5.7	4.4	4.6
** Native Hawaiian** ^¶^				
Early-stage	65.2	34.3	21.2	48.4
Late-stage	32.6	60.8	74.2	48.4
Unknown	2.2	5.0	4.5	—**
** Pacific Islander** ^¶^				
Early-stage	55.7	36.5	18.9	40.0
Late-stage	40.3	56.7	76.9	54.7
Unknown	4.0	6.7	4.3	5.3

**Abbreviation**: NOS = not otherwise specified.

* Early-stage (localized) cancer is confined to the primary site, and late-stage (regional or distant stage) cancer has spread to lymph nodes or other parts of the body. Cases identified only through autopsies or death certificates were excluded from the stage analyses.

^†^ Cancer incidence data were compiled from cancer registries that meet the data quality criteria for all invasive cancer sites combined, representing 99% of the U.S. population.

^§^ The current analysis is restricted to persons who reported non-Hispanic ethnicity.

^¶^ Groups were combined as follows: Other Southeast Asian includes Cambodian, Hmong, Laotian, and Thai persons; South Asian includes Asian Indian, Pakistani, and Asian Indian or Pakistani race NOS persons; Pacific Islander includes Chamoru, Fiji Islander, Guamanian race NOS, Melanesian race NOS, Micronesian race NOS, Papua New Guinean, Polynesian race NOS, Samoan, Tahitian, Tongan, and Pacific Islander race NOS persons; Other Asian includes data for persons whose race was not further specified or who are members of racial groups that did not include the other 24 Asian American, Native Hawaiian, or other Pacific Islander subgroups.

** Counts were suppressed because fewer than six cases were reported.

## Discussion

Persons of Asian and NHPI origin are often aggregated into one racial group (*2*,*3*); however, the findings in this report show differences in cancer distribution and late-stage cancer diagnoses among Asian and NHPI subgroups. These results are generally consistent with a study that found a higher percentage of distant-stage colorectal cancers among men with an origin in Cambodia, Laos, or Vietnam (*6*). Late-stage cancer cases can be attributed in part to disparities in cancer screening (*7*). National Health Interview Survey data from 2018 show that Asian American persons were less likely than non-Hispanic White or non-Hispanic Black or African American persons to be up to date with colorectal cancer testing, pap smear, or mammogram (*7*,*8*). One way CDC addresses cancer disparities is with the development of resources such as the Breast Cancer Disparities Tool Kit.*** Although this online tool is not tailored to specific populations, it encourages coordinated partner engagement, sustainable implementation from trusted messengers, and evaluation to address social determinants of health and reduce mortality among groups that experience breast cancer disparities.

Ongoing surveillance is important in addressing and evaluating cancer disparities among different populations. An evaluation of the impact of COVID-19 on the number of breast and cervical cancer screening tests provided through CDC’s National Breast and Cervical Cancer Early Detection Program found that in April 2020, breast cancer screening among Asian and NHPI women declined 97% compared with the previous 5-year average; cervical cancer screening decreased by 92% (*8*). To help address the decline in screening among certain populations, CDC has partnered with health care providers to resume timely use of preventive tests such as cancer screening (*8*). Cancer screening tests can aid in the early detection of breast, cervical, colorectal, and lung cancers, when treatment is likely to be most effective (*8*).

A better understanding of cancer distribution among Asian and NHPI persons can support the development of tailored cancer prevention and control initiatives. For example, in response to studies that found high rates of liver cancer among Asian and NHPI persons combined (*9*), the Hawaii Comprehensive Cancer Coalition developed a culturally and linguistically appropriate statewide hepatitis B vaccination media campaign^†††^ for non–U.S.-born Asian and NHPI persons. The Massachusetts Comprehensive Cancer Steering Committee is working to increase breast cancer screening rates among Asian women by collaborating with advocacy and state outreach partners.^§§§^ Culturally and linguistically competent programs might help address disparities in cancer incidence and outcomes; such programs are particularly well-positioned to succeed when they consider social determinants of health (i.e., social and environmental circumstances in which persons live, learn, work, and play^¶¶¶^) (*10*).

The findings in this report are subject to at least four limitations. First, current national population denominators were not available for all subgroups; therefore, comparing rates was not possible. Second, because of small case counts among certain subgroups, comparisons between certain subgroups were limited. Third, multiracial identification was not included in this analysis. Finally, other risk factors not routinely collected by cancer registries could not be assessed.

Differences in cancer distribution among Asian and NHPI subgroups exist. Using population-based cancer registries to identify groups with disproportionate cancer outcomes might help guide the design and implementation of cancer prevention and control programs that consider social determinants of health. CDC funds several national cancer programs that are required to include activities to identify and address drivers of cancer health disparities.****

**Summary**

**What is already known about this topic?**

Non-Hispanic Asian and non-Hispanic Native Hawaiian and Pacific Islander (NHPI) persons represent a growing segment of the U.S. population, and are often aggregated in analyses.

**What is added by this report?**

Cancer incidence among 25 Asian and NHPI subgroups differed by sex, age, cancer type, and stage at diagnosis. For example, lung cancer was the most common cancer among Chamoru, Micronesian, and Vietnamese persons; colorectal cancer was the most common cancer among Cambodian, Hmong, Laotian, and Papua New Guinean persons.

**What are the implications for public health practice?**

Understanding cancer distribution among Asian and NHPI subgroups might help guide development and implementation of culturally and linguistically relevant programs addressing health disparities and social determinants of health.

## Footnotes

*
https://www.cdc.gov/cancer/uscs

†
https://apps.who.int/iris/bitstream/handle/10665/96612/9789241548496_eng.pdf

§ Cancer registries use uniform data items and codes as documented by the North American Association of Central Cancer Registries (NAACCR). This analysis used Race 1 variable, which is defined in the NAACCR Data Standards and Data Dictionary, Volume II, Version 21, Chapter X. https://www.naaccr.org/data-standards-data-dictionary

¶ The Asian group includes East Asian (Chinese, Japanese, and Korean), Southeast Asian (Cambodian, Filipino, Hmong, Laotian, Thai, and Vietnamese), South Asian (Asian Indian, Pakistani, and Asian Indian or Pakistani NOS), and Other Asian. The NHPI group includes NHPI (Chamoru, Fiji Islander, Guamanian NOS, Melanesian NOS, Micronesian NOS, Native Hawaiian, Pacific Islander NOS, Papua New Guinean, Polynesian NOS, Samoan, Tahitian, and Tongan). Persons who were members of racial subgroups not included in the 24 subgroups or whose race was not specified further than “Asian subgroup” were combined into the Other Asian group. Because of low case counts, in some analyses, Cambodian, Laotian, Hmong, and Thai persons were combined into the Other Southeast Asian group.

**
https://www.cdc.gov/cancer/uscs/dataviz/index.htm

††
https://www.cdc.gov/cancer/dcpc/prevention/screening.htm

§§ Early-stage was defined as a tumor being confined to the organ of origin without extension beyond the primary organ. Late-stage was defined as a direct extension of the tumor to adjacent organs, structures, or spread to regional lymph nodes or to parts of the body remote from the primary tumor.

¶¶ 45 C.F.R. part 46.102(l)(2), 21 C.F.R. part 56; 42 U.S.C. Sect. 241(d); 5 U.S.C. Sect. 552a; 44 U.S.C. Sect. 3501 et seq.

***
https://www.cdc.gov/cancer/breast/what_cdc_is_doing/

†††
https://ftp.cdc.gov/pub/Publications/Cancer/ccc/hawaii_ccc_plan-508.pdf

§§§
https://ftp.cdc.gov/pub/Publications/Cancer/ccc/massachusetts_ccc_plan-508.pdf

¶¶¶
https://health.gov/healthypeople/priority-areas/social-determinants-health

**** https://www.cdc.gov/media/releases/2022/p0608-cancer-award.html; https://www.cdc.gov/cancer/health-equity/
